# High-Throughput Methods to Detect Long Non-Coding RNAs

**DOI:** 10.3390/ht6030012

**Published:** 2017-08-31

**Authors:** Shizuka Uchida

**Affiliations:** Cardiovascular Innovation Institute, University of Louisville, 302 E Muhammad Ali Blvd, Louisville, KY 40202, USA; heart.lncrna@gmail.com; Tel.: +1-502-854-0570; Fax: +1-502-852-7195

**Keywords:** long non-coding RNA, non-coding RNA, microarray, RNA-seq

## Abstract

Increasing evidence suggests that the numbers of long non-coding RNAs (lncRNAs) are more than those of protein-coding genes in various organisms. Although the detection methods for lncRNAs are being increasingly established, there are advantages and disadvantages that exist for each method. In this opinion article, I highlight the differences between microarrays and RNA sequencing (RNA-seq) for the detection of lncRNAs. Compared to RNA-seq, microarrays are limited to the known sequences. However, the detection method as well as data analysis workflow is more established, which makes it easier to analyze the data for bench scientists without extensive knowledge about computer programming. In order to highlight the usage of microarrays over RNA-seq for the detection of lncRNAs, we are organizing a special issue for *High-Throughput* called “Microarrays in Non-Coding RNAs Profiling”, which will include the specific usages of microarrays for lncRNAs.

It is now firmly recognized that coding parts of protein-coding genes occupy a very minor part of the mammalian genome, although a vast majority of the mammalian genome is transcribed to RNA [1,2,3]. Currently, those RNAs that do not encode for proteins are collectively called “non-coding RNAs (ncRNAs)”, which are further categorized by their lengths rather than their functionalities. Although the functions of small RNAs, including microRNAs (miRNAs), have been elucidated in the past two decades, potential functions of longer ncRNAs are understudied and just recently being investigated in various fields of study. Among longer ncRNAs, those longer than 200 nucleotides are classified as “long non-coding RNAs (lncRNAs)”, which were once considered “junk DNA” [4]. As technology advances, more and more lncRNAs are discovered and reported, although their functions remain mostly unknown. Although their functions are unknown, it is speculated that the number of lncRNAs increases as organisms move up in the evolutionary ladder [5]. Thus, understanding lncRNAs might shed light on the complexity of organisms during evolution [6,7,8,9,10,11,12,13]. Also, lncRNAs are involved in a variety of cellular processes [14,15,16,17,18,19,20] and their dysregulations are linked to some diseases [21,22,23,24,25,26]. 

To detect lncRNAs, there are two most common methods: microarrays and RNA sequencing (RNA-seq) using next generation sequencers. The former is well-established method, which has a rather long history for the detection of protein-coding genes. Given that probes can be designed as long as sequences are known, it is not surprising that microarrays can be designed to detect lncRNAs. More importantly, the previously built microarrays contain probe sequences that match lncRNAs, although they were initially designed for protein-coding genes. This is due partially to the re-annotation of previously thought protein-coding genes as lncRNAs. Recently, we and others re-annotated the probe sequences of microarrays and found that many lncRNAs can be recorded from Affymetrix-based GeneChips as these types of microarrays contain many probe sequences that do not match exons of protein-coding genes [27,28]. In the case of RNA-seq, unbiased genome-wide screening of lncRNAs is possible. Currently, there are two main methods of generating a sequencing library for RNA-seq experiments. One is to use oligo dT beads to enrich mRNAs with poly A tails. Another is to deplete ribosomal RNAs (rRNAs) from the total RNA to enrich for RNAs that are not rRNA as rRNA constitutes ~80% of total RNA followed by 15% transfer RNAs (tRNAs) and only 5% for all other RNAs, including protein-coding genes and lncRNAs [29]. The former method will result in the identification of protein-coding genes and lncRNAs with poly A tails (~60% of total lncRNAs) [30], while the latter can identify the rest of lncRNAs and a newly emerging class of lncRNAs called “circular RNAs (circRNAs)” [31,32,33,34], in addition to those identified in the former method. The presence of circRNAs is only detected with the latter method as circRNAs arise from exons and/or introns that are spliced out, which are devoid of poly-A tails. Compared to microarrays, the data analysis of RNA-seq is still a matter of debate as there are many algorithms available to normalize the data and to quantify the transcripts, for which no one method is superior over others [35,36,37,38]. Furthermore, there is an ongoing debate about the quantification of RNA-seq reads (e.g., RPKM (Reads Per Kilobase Million), FPKM (Fragments Per Kilobase Million), and TPM (Transcripts Per Kilobase Million)), which makes the RNA-seq data analysis complicated compared to that of microarrays.

Although RNA-seq offers more comprehensive coverage of whole transcriptomes compared to microarrays, there is a question of how deep the RNA-seq reads should be. It is commonly accepted that at least 10–20 million reads are needed to be comparable with the expression profiling performed via microarrays [39]. However, this is based on the poly-A-based RNA-seq, which cover protein-coding genes and ~60% of lncRNAs but not the remaining lncRNAs without poly-A tails and circRNAs. Thus, it is not as comprehensive coverage of whole transcriptome one would expect. Given such situation, microarrays may perform better for surveying the known sequences. It is absolutely true that unknown (and novel) lncRNAs will be discovered via RNA-seq than microarrays. However, a caution is needed as many of such novel lncRNAs contain many repetitive elements as we recently reported [40]. Furthermore, the mapping rate of sequence reads depends on the version of reference genome, such that the most recent human genome assembly GRCh38/hg38 has much better mapping rate than GRCh37/hg19 [40]. In addition, the computational power needed to analyze RNA-seq data is much more demanding than that of microarrays. Furthermore, the involvement of experienced bioinformaticians is required to analyze RNA-seq data properly, which is not the case for microarray data analysis as this technology is much more mature than RNA-seq and many well-established software and bioinformatics tools are available for microarray data analysis.

One additional point about the difference between microarrays and RNA-seq is that, in most cases, microarrays do not involve PCR-based amplification of sample probes (especially in the case of gene/exon arrays of Affymetrix), whereas RNA-seq requires multiple cycles of PCR amplifications. Given that PCR amplifications might introduce a certain bias, it may skew the data for GC-rich regions. Taken together, although RNA-seq is more powerful on detecting novel lncRNAs as well as splicing and RNA modifications (e.g., A-to-I RNA editing [41,42]), if one is interested in finding out about known signaling pathways and lncRNAs (although not many), it is better off using microarrays than RNA-seq as the protocol and data analysis are more established. In order to highlight the usage of microarrays over RNA-seq for the detection of lncRNAs, we are organizing a special issue for *High-Throughput* called “Microarrays in Non-Coding RNAs Profiling”, which will include the specific usages of microarrays for lncRNAs.
